# Stem Cell and EV/sEV Strategies for Neuroimmune Niche Reprogramming in Selected CNS Disorders: Mechanisms, Engineered Delivery, and Translational Readiness

**DOI:** 10.1002/cns.71211

**Published:** 2026-10-21

**Authors:** Wenjing Lu, Junqiang Yan, Hongfang Feng

**Affiliations:** ^1^ The First Affiliated Hospital, College of Clinical Medicine Henan University of Science and Technology Luoyang Henan China

**Keywords:** aging, immunosenescence, mitophagy, neuroinflammation, reactive oxygen species, redox signaling fidelity, systems biology

## Abstract

**Background:**

Aging has traditionally been interpreted through the accumulation of oxidative damage. However, reactive oxygen species (ROS) also act as regulated signals that support immune activation, metabolic adaptation, stress resistance, inflammatory resolution, and cell‐fate decisions. We define redox signaling fidelity as the accuracy with which a specified physiological input is converted into a source‐specific, spatially confined, temporally patterned, chemically reversible, proportionately decoded, and appropriately resolved redox response.

**Methods:**

This critical narrative review develops redox signaling fidelity as a testable systems framework rather than as a synonym for oxidative stress, redox homeostasis, oxidative eustress, hormesis, the redox code, or Redox‐stress Response Capacity. The framework organizes redox information flow into five linked layers: generation, encoding, transmission, decoding, and resolution/reset. Aging may lower fidelity through source uncoupling, persistent cysteine oxidation, microdomain leakage, decoder bias, and defective autophagic, mitophagic, lysosomal, or proteostatic reset. Immune aging and the aging brain are used as cross‐scale test cases in which physiological ROS pulses must be distinguished from chronic redox noise. We propose operational readouts, including source coupling, redox signal‐to‐noise ratio, reversibility, spatial leakage, decoder proportionality, mutual information, and resolution kinetics, and map these readouts to single‐cell imaging, redox proteomics, spatial and single‐cell omics, and dynamic modeling.

**Conclusions:**

The fidelity framework predicts that lowering bulk ROS is not sufficient to restore redox function. More decisive studies should pair controlled inputs with compartment‐resolved redox dynamics, reversible molecular encoding, pathway output, and functional recovery in the same young‐versus‐aged system. Interventions should be evaluated by whether they reduce pathological redox noise while preserving physiological pulses, spatial routing, adaptive decoding, and timely resolution.

## Graphical abstract

Stem cell‐ and EV/sEV‐based interventions may reprogram the CNS neuroimmune niche by suppressing glial inflammation, enhancing trophic and synaptic support, stabilizing BBB communication, and improving metabolic resilience. Clinical translation will require mechanism‐linked potency assays, MISEV‐aligned EV reporting, biodistribution, GMP manufacturing, and biomarker‐guided trials.

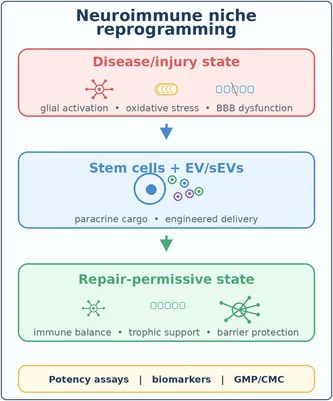

## 1 Introduction

Selected central nervous system (CNS) disorders, including Alzheimer's disease (AD), Parkinson's disease (PD), multiple sclerosis (MS), amyotrophic lateral sclerosis (ALS), Huntington's disease (HD), frontotemporal dementia (FTD), and traumatic brain injury (TBI), evolve within a complex tissue environment rather than through neuronal injury alone. Disease‐specific triggers such as amyloid and tau pathology, alpha‐synuclein aggregation, demyelination, motor neuron vulnerability, mutant huntingtin toxicity, TDP‐43 pathology, or mechanical injury converge on chronic neuroinflammation, glial‐state remodeling, mitochondrial dysfunction, oxidative stress, impaired autophagy, and blood–brain barrier (BBB) disturbance [1, 2, 3, 4, 5, 6, 7, 8, 9].

Most approved treatments for these disorders remain symptomatic or partially disease‐modifying. Dopaminergic replacement and deep brain stimulation improve selected PD symptoms; cognition‐directed drugs and anti‐amyloid strategies target specific AD biology, and immunomodulatory regimens reduce inflammatory activity in MS; however, these approaches rarely restore lost circuitry or durably reset the inflammatory and metabolic tissue state that sustains disease progression [10, 11, 12]. This therapeutic gap has stimulated interest in biological strategies that combine neuroprotection, immune modulation, trophic support, and repair‐permissive niche remodeling.

Stem cells and stem cell‐derived products have been investigated in this setting because they can influence multiple pathological nodes. Mesenchymal stromal cells (MSCs), neural stem or progenitor cells (NSCs/NPCs), pluripotent‐stem‐cell‐derived differentiated neural products, dental pulp‐derived MSC‐like cells, and extracellular vesicle (EV/sEV) preparations differ markedly in source, administered format, potency, manufacturing complexity, immunogenicity, and translational maturity. Across models, many reported effects are better explained by paracrine signaling, EV/sEV‐mediated cargo transfer, immune regulation, and trophic support than by durable engraftment or broad neuronal replacement [13, 14, 15, 16, 17, 18, 19, 20, 21, 22, 23, 24].

Engineered delivery systems, including biomaterials, nanocarriers, cell‐tracking platforms, and EV/sEV engineering, may help address poor homing, low retention, difficult BBB delivery, insufficient target engagement, and incomplete product monitoring [25, 26, 27, 28, 29]. These technologies should be viewed as tools for improving precision, reproducibility, and pharmacological interpretability, not as independent proof that stem cell products are clinically effective.

Accordingly, this review adopts a neuroimmune niche reprogramming framework. In this framework, therapeutic value is judged by the capacity of stem cell and EV/sEV products to reshape glial activation, peripheral immune communication, BBB function, oxidative and metabolic stress, senescence‐associated inflammatory signaling, trophic support, and repair‐permissive tissue states. Cell replacement remains important in selected contexts, particularly focal neuronal loss in PD, but it should not be generalized across diffuse or multifactorial CNS disorders without direct evidence of survival, integration, connectivity, and functional causality.

## 2 Conceptual Definition and Operational Criteria

Neuroinflammation describes a pathological process, whereas immunomodulation describes an intervention‐induced change in immune signaling. We use neuroimmune niche reprogramming more narrowly to denote a coordinated state transition of the tissue ecosystem that couples resident and infiltrating immune‐cell states to blood–brain barrier and perivascular function, mitochondrial and immunometabolic fitness, trophic and synaptic support, and extracellular or structural repair signals. A decrease in one cytokine or a binary M1/M2 or A1/A2 shift is therefore insufficient by itself to establish niche reprogramming.

Operationally, the framework asks four questions: whether the effect is linked to a defined mechanism rather than a nonspecific stress response; whether it engages interacting niche compartments or demonstrates causal cross‐compartment communication; whether the state change persists long enough after product clearance to influence tissue function; and whether perturbation of the proposed pathway abolishes target engagement or functional benefit. Early studies may not satisfy every criterion, but the term reprogramming should be reserved for evidence that goes beyond transient immunosuppression.

This review differs from prior reviews that primarily catalog stem cell trials or engineered exosome platforms [23]. We organize the field around mechanism‐defined neuroimmune niche reprogramming, explicitly separate cell source from administered therapeutic format, distinguish cell replacement from paracrine niche modulation, align EV terminology with MISEV2023 principles, add a quantitative clinical‐evidence table (Table 2), and emphasize translational criteria such as potency assays, biodistribution, biomarkers, GMP manufacturing, dose–response relationships, and regulatory classification. This framing is intended to make the manuscript more suitable for CNS therapeutics and translational neuroscience readerships. The overall conceptual framework is summarized in Figure 1.

**FIGURE 1:**
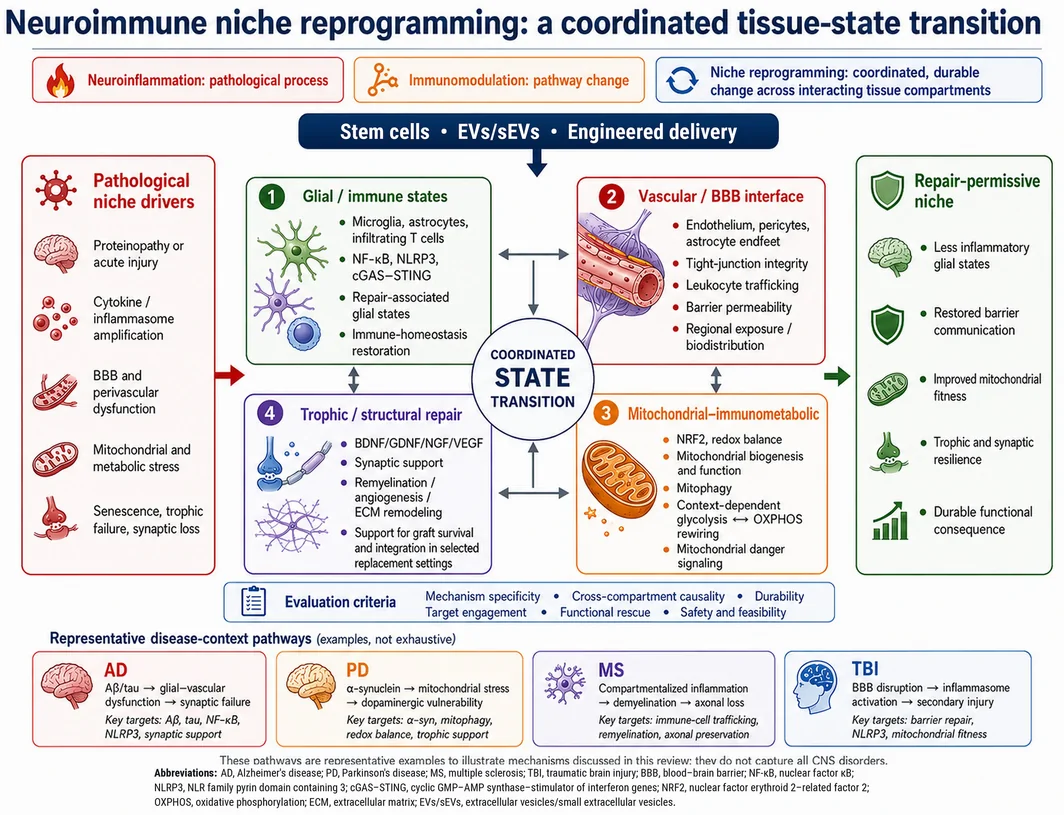
Neuroimmune niche reprogramming as a coordinated tissue‐state transition. The framework distinguishes neuroinflammation (a pathological process) from immunomodulation (an intervention‐induced pathway change) and reserves niche reprogramming for coordinated, mechanism‐linked, and sufficiently durable changes across four interacting compartments: glial/immune states, vascular and blood–brain barrier function, mitochondrial–immunometabolic fitness, and trophic/structural repair. Operational evidence should include mechanism specificity, cross‐compartment causality, durability, target engagement, and functional rescue. Disease‐context examples include Aβ/tau‐associated glial–vascular dysfunction in Alzheimer's disease, α‐synuclein‐associated mitochondrial stress in Parkinson's disease, compartmentalized inflammatory demyelination in multiple sclerosis, and BBB–inflammasome‐driven secondary injury in traumatic brain injury. AD, Alzheimer's disease; BBB, blood–brain barrier; cGAS–STING, cyclic GMP–AMP synthase–stimulator of interferon genes; ECM, extracellular matrix; EVs, extracellular vesicles; MS, multiple sclerosis; NF‐κB, nuclear factor κB; NLRP3, NLR family pyrin domain containing 3; NRF2, nuclear factor erythroid 2–related factor 2; OXPHOS, oxidative phosphorylation; PD, Parkinson's disease; sEVs, small extracellular vesicles; TBI, traumatic brain injury.

The added value of this review is therefore not another inventory of candidate products. Instead, it provides a disease‐independent decision framework that asks whether a given intervention has a biologically appropriate mechanism, a reproducible product definition, demonstrable CNS exposure and target engagement, and a feasible regulatory and clinical development pathway. This approach also permits positive, negative, and neutral trials to be interpreted within the same translational logic.

The disease‐specific synthesis focuses on seven representative CNS contexts selected to span chronic neurodegeneration (AD, PD, FTD, ALS, and HD), immune‐mediated demyelination (MS), and acute/subacute neuroimmune injury (TBI). Evidence from ischemic stroke, spinal cord injury, subarachnoid hemorrhage, and optic nerve injury is used selectively to support cross‐cutting mechanistic or delivery principles rather than as an equivalent disease‐level therapeutic appraisal.

## 3 Literature Search and Selection Strategy

This is a narrative translational review. The final literature‐search window covered English‐language articles published mainly from January 1, 2010 through June 30, 2026 in PubMed, Web of Science, Scopus, and ClinicalTrials.gov. June 30, 2026 was used as the final literature cutoff for the second revision. Official regulatory webpages were also consulted to verify current authorization status; their access dates are reported separately in the reference list and do not alter the literature cutoff. Search terms included combinations of: stem cell, mesenchymal stromal cell, neural stem cell, neural progenitor cell, induced pluripotent stem cell, embryonic stem cell, dental pulp stem cell, extracellular vesicle, small extracellular vesicle, exosome, neuroimmune, microglia, astrocyte, inflammasome, NLRP3, blood–brain barrier, senescence, Alzheimer's disease, Parkinson's disease, amyotrophic lateral sclerosis, multiple sclerosis, Huntington's disease, frontotemporal dementia, traumatic brain injury, ischemic stroke, spinal cord injury, subarachnoid hemorrhage, optic nerve injury, biomaterial, nanocarrier, potency assay, biomarker, GMP, and clinical trial. Database‐specific search strings are provided in Data S1 to improve reproducibility.

We prioritized original mechanistic studies, animal studies with defined therapeutic endpoints, peer‐reviewed clinical trials, regulatory or guideline documents, and high‐quality reviews that directly informed mechanism, delivery, manufacturing, or clinical translation. For clinical evidence, we prioritized primary human studies involving direct administration of a stem/progenitor‐derived cellular product or EV/sEV preparation in a CNS indication, together with regulatory records that materially altered translational status. Registered but unpublished studies were distinguished from peer‐reviewed clinical reports and were not treated as efficacy evidence. Studies were excluded from claim‐specific support when they focused primarily on non‐CNS indications, lacked sufficient mechanistic or translational relevance, duplicated information covered by higher‐quality sources, or used nonstandard terminology without adequate characterization. Because this is not a systematic review, no quantitative meta‐analysis was performed; evidence was synthesized according to mechanism, disease context, product type, and translational readiness.

## 4 Therapeutic Source Categories and Administered Product Formats

Therapeutic source and administered product format should be separated conceptually. Source‐derived cellular categories differ in lineage potential, biological function, and manufacturing constraints, whereas living cells, secretome/EV preparations, and engineered combination products represent distinct administered formats. Their therapeutic relevance depends on dominant disease mechanism, route, duration of action, and feasibility of standardized manufacturing. Table 1 therefore compares these two dimensions separately rather than placing cell sources and EV/sEV product formats on one taxonomic level.

**TABLE 1:** Two‐dimensional classification of therapeutic source categories and administered product formats for neuroimmune niche modulation.

Panel A. Source‐derived therapeutic cell categories			
Feature	MSC‐derived products (including DPSC subgroup)	Neural stem/progenitor products	Pluripotent‐stem‐cell‐derived differentiated neural products (iPSC/hESC)
Primary therapeutic logic	Paracrine immunomodulation, trophic/barrier support, niche modulation [30, 31, 32]. DPSCs are treated as a neural‐crest‐derived MSC‐like subgroup.	Local neuroprotection, trophic support, selected remyelination/repair, and lineage contribution [33, 34, 35].	Lineage‐specified neuronal replacement. iPSC disease modeling/drug screening is an upstream research use, not an administered therapeutic product [36, 37, 38, 39].
Clinical maturity	Broadest CNS cell‐product experience; safety established in several AD/MS/PD/TBI studies, but efficacy is inconsistent [30, 40, 41, 42, 43, 44].	Early clinical testing in progressive MS and ALS; feasibility/safety stronger than efficacy [33, 45].	PD replacement has early human safety/target‐engagement evidence; raguneprocel received conditional/time‐limited authorization in Japan in 2026 [36, 37, 46, 47].
Major risks	Donor/source and passage heterogeneity, variable potency, limited persistence, batch variability [30, 42, 43, 44].	Survival, differentiation control, ectopic growth, graft–host interactions, invasive delivery [33, 34, 45].	Genomic instability, residual pluripotency, tumorigenicity, immunogenicity, lineage purity, long‐term surveillance [36, 37, 48].
Best‐fit CNS context	Inflammation‐dominant or barrier‐injury states; adjunctive immune/trophic modulation [30, 31, 32, 49, 50].	Focal injury/repair, selected remyelination, and local trophic support [33, 34, 35].	Anatomically focal neuronal loss, especially dopaminergic replacement in PD [36, 37].
Reproducibility‐critical variables	Donor age/comorbidity, tissue/tooth source, passage, senescence, culture conditions, cryopreservation, site/operator effects [30, 43, 44, 48].	Lineage composition, expansion history, maturation state, post‐thaw viability, graft‐to‐graft consistency [33, 36, 37, 45].	Clone/reprogramming method, genomic integrity, differentiation batch, residual pluripotency, lineage purity and maturation [36, 37, 48].
Key development need	Mechanism‐linked potency assay, GMP‐compatible release criteria and lot comparability [30, 42, 43, 44, 48].	Survival/integration monitoring and functional causality [33, 36, 37, 45].	Lineage purity, genomic safety, maturation, immune strategy and long‐term surveillance [36, 37, 48].

Panel B. Administered product format (independent of cell source)			
Feature	Living‐cell products	Secretome/EV/sEV preparations	Engineered/combination products
Administered material	Viable cells/progenitors with paracrine and/or replacement activity.	Cell‐free secretome or defined EV/sEV preparation derived from a parent‐cell population.	EV engineering, nanocarriers, hydrogels/scaffolds, cell‐attached materials, or other combination systems.
Principal advantage	Multicomponent biological activity; possible local persistence and selected cell replacement.	Repeatable cell‐free dosing; avoids uncontrolled cell proliferation; cargo can be characterized/engineered.	Can improve targeting, retention, local exposure, tracking, or controlled release.
Main limitation	Donor/lot variability, viability, persistence, ectopic growth/immune/procedural risks.	Cargo and particle heterogeneity; incomplete PK/PD; systemic clearance; no FDA‐approved exosome products [51, 52, 53, 54, 55].	Added engineering creates new CQAs, purification steps, toxicity/off‐target risks, and regulatory burden.
Dose/PK considerations	Cells/kg or cells/site; viability and tissue persistence must be measured.	Particle number, protein and producer‐cell equivalent are not interchangeable; quantitative biodistribution and mechanism‐linked activity are needed.	Head‐to‐head comparison should demonstrate a prespecified gain in exposure, retention, dose reduction, potency, or feasibility.
Manufacturing/release	Identity, viability, purity, sterility, potency, stability, and comparability after process changes.	MISEV‐aligned characterization plus contaminants, cargo fingerprint, stability, dose metrics, and recipient‐cell potency.	Platform‐specific CMC, component comparability, and combination‐product/device requirements.
Best‐fit use	When sustained local biological activity or lineage‐specific replacement is required.	When repeatable cell‐free modulation is desired and live‐cell risks are unacceptable.	Only when the least‐complex adequate platform does not achieve required exposure/retention/monitoring.

*Note:* Cell/source category and administered product format are intentionally separated. DPSCs are treated as a neural‐crest‐derived MSC‐like subgroup; iPSC/hESC therapies are classified by the differentiated cell product administered; EV/sEV preparations may be derived from multiple parent‐cell types.

Abbreviations: CMC, chemistry, manufacturing, and controls; CQAs, critical quality attributes; DPSC, dental pulp stem cell; EV, extracellular vesicle; GMP, good manufacturing practice; hESC, human embryonic stem cell; iPSC, induced pluripotent stem cell; MSC, mesenchymal stromal cell; NSC/NPC, neural stem/progenitor cell; PK/PD, pharmacokinetics/pharmacodynamics; sEV, small extracellular vesicle.

### 4.1 Mesenchymal Stromal Cells

Bone marrow‐, adipose‐, umbilical cord‐, and other tissue‐derived MSCs have been widely investigated because their principal activity is paracrine and immunoregulatory rather than replacement‐based. MSC‐derived secreted factors and EVs/sEVs can influence microglia, astrocytes, endothelial cells, and peripheral immune responses, and their activity is increasingly interpreted through secretome‐mediated rather than replacement‐based mechanisms [56]. In TBI, ischemia, spinal cord injury, PD, and AD models, MSCs or MSC‐derived EVs/sEVs have been associated with reduced inflammatory amplification, modulation of NLRP3/NF‐κB‐related pathways, improved barrier integrity, and partial functional recovery [30, 31, 32, 57, 58, 59, 60, 61, 62, 63, 64, 65, 66].

Limited long‐term engraftment does not necessarily contradict biological activity. Many MSC effects are plausibly mediated by transient secretory signaling, immune‐cell education, mitochondrial or oxidative stress modulation, and improved repair‐permissive tissue states. The main translational challenge is therefore not to prove that MSCs become neurons, but to define product identity, potency, release criteria, biodistribution, dosing, and target‐engagement biomarkers.

### 4.2 Dental Pulp Stem Cells

Dental pulp stem cells (DPSCs) are neural crest‐derived MSC‐like stromal cells obtained mainly from permanent teeth or exfoliated deciduous teeth. In the present classification they are treated as a source‐specific MSC‐like subgroup rather than as a taxonomically equivalent category parallel to all MSCs [67, 68]. CNS‐relevant preclinical studies support trophic, anti‐inflammatory, microglial, pyroptotic, and neural‐repair effects in selected spinal cord injury, subarachnoid hemorrhage, stroke, and microglial models [69, 70, 71, 72, 73, 74, 75, 76]. Their advantages include relatively noninvasive procurement and potential banking, whereas major barriers include donor age, tooth source, culture history, product heterogeneity, and limited clinical maturity.

### 4.3 Neural Stem/Progenitor Cells

NSCs and NPCs have a stronger conceptual link to neural repair because they can differentiate toward neuronal and glial lineages and secrete trophic or immunomodulatory factors. Their translational rationale is strongest when local delivery, structural support, or reconstruction of a focal CNS niche is required. However, survival, controlled differentiation, integration, ectopic growth, and immunological compatibility remain major constraints. Preclinical studies of NSC/NPC delivery and repair, together with early progressive‐MS clinical experience, support feasibility and biological plausibility, but definitive clinical efficacy remains unproven [33, 34, 35, 77, 78, 79, 80, 81, 82].

### 4.4 Induced Pluripotent Stem Cells

Induced pluripotent stem cells (iPSCs) are valuable upstream platforms for human disease modeling and drug screening and can also serve as sources for lineage‐specified therapeutic cell products. iPSC‐derived neurons, astrocytes, and microglia can model genotype‐specific inflammatory responses in AD, ALS/FTD, PD, and other disorders [38, 39, 62, 83, 84, 85, 86, 87, 88]. Therapeutic transplantation should, however, be classified by the differentiated product that is administered rather than by the undifferentiated iPSC platform. In PD, differentiated dopaminergic progenitor products derived from both iPSCs and hESCs have reached clinical testing [36, 37]. Translation requires rigorous control of genomic stability, residual pluripotency, lineage purity, maturation state, immunogenicity, and tumorigenicity.

Overall, source‐derived therapeutic categories are best distinguished as MSC‐derived products (including source‐specific MSC‐like subgroups such as DPSCs), neural stem/progenitor products, and pluripotent‐stem‐cell‐derived differentiated neural products. Separately, administered formats include living‐cell products, secretome/EV/sEV preparations, and engineered or combination products. EV/sEVs may be generated from several parent‐cell categories and should therefore not be treated as a cell source equivalent to MSCs or iPSCs. Source and format should be selected according to disease mechanism and intended clinical endpoint rather than presumed regenerative hierarchy.

No platform consistently outperforms all others. MSC‐derived products and EV/sEV preparations are most compatible with spatially distributed inflammatory or barrier‐related pathology, but transient exposure and product heterogeneity may limit durability. NSCs/NPCs occupy an intermediate position, combining local trophic activity with selected lineage contribution, whereas pluripotent‐stem‐cell‐derived differentiated neural products offer the clearest replacement rationale for anatomically focal neuronal loss but impose the highest demands for lineage purity, genomic stability, immunological management, and long‐term surveillance. Platform selection should therefore be based on mechanistic fit and developability rather than on a presumed hierarchy of regenerative capacity.

## 5 Stem Cell‐Derived EVs/sEVs as Cell‐Free Neuroimmune Niche Modulators

### 5.1 Terminology and MISEV2023‐Aligned Reporting

In this review, we use extracellular vesicles (EVs) or small extracellular vesicles (sEVs) unless the cited study provides sufficient evidence for endosomal biogenesis. This terminology follows MISEV2023, which recommends defining EV preparations according to operational characteristics such as size, density, biochemical markers, cell source, separation method, and characterization profile rather than assuming exosomal origin [53].

For CNS translation, EV/sEV studies should report parent‐cell source, culture conditions, preconditioning, separation method, particle number, protein amount, EV markers and non‐EV contaminants, cargo profiling, storage conditions, dosing metrics, route of administration, biodistribution, and potency readouts. Without these details, therapeutic claims are difficult to compare across laboratories or to move into GMP manufacturing.

### 5.2 EV Heterogeneity as a Mechanistic and Product Variable

EVs are not a single molecular species but overlapping populations that differ in size, density, membrane composition, biogenesis, surface proteins, organellar content, and cargo [89]. Differential ultracentrifugation, size‐exclusion chromatography, precipitation, filtration, and affinity capture enrich distinct and only partially overlapping fractions; consequently, two preparations generated from the same producer cells may not be biologically equivalent. Proteomic and functional fractionation studies further show that canonical markers and active cargo are unevenly distributed among EV subtypes [53, 90, 91, 92].

This heterogeneity is biologically meaningful rather than merely analytical noise. Producer‐cell lineage, donor, passage, inflammatory or hypoxic conditioning, metabolic state, and collection interval can alter vesicle composition and target‐cell tropism. A therapeutic program must therefore state whether the intended product is the complete heterogeneous preparation, an enriched functional subfraction, or an engineered vesicle class; activity observed in bulk particles should not be attributed to a named subtype without fraction‐specific evidence.

Mechanistic and release testing should combine orthogonal size and concentration measurements, EV‐enriched and non‐EV contaminant markers, density or affinity information when relevant, cargo profiling, and a recipient‐cell potency assay. Single‐vesicle or high‐resolution analyses can be used during development to identify active or potentially harmful subpopulations, but clinical release methods must remain robust and scalable. Standardization should therefore control a reproducible range of heterogeneity rather than assume that all particles are interchangeable [53, 90, 91, 92].

### 5.3 EV/sEV BBB Interaction and Intranasal Nose‐to‐Brain Delivery

Stem cell‐derived EVs/sEVs can protect regulatory cargo within a lipid bilayer and mediate communication among neural, glial, endothelial, and immune cells. After systemic administration, EV/sEV interaction with the BBB may involve endothelial binding, adsorptive or receptor‐mediated uptake/transcytosis, and inflammation‐dependent changes in barrier permeability; however, CNS penetration should not be assumed as a class effect and requires quantitative, product‐specific biodistribution and dose–response assessment [52].

Intranasal administration is mechanistically distinct from systemic BBB transcytosis. Nose‐to‐brain access may occur through olfactory and trigeminal pathways and can therefore partially bypass the conventional blood‐to‐brain endothelial barrier. Intranasal delivery should consequently be analyzed as a route‐specific CNS access mechanism rather than listed as another form of BBB transcytosis [25, 28, 59, 93].

### 5.4 Regulation of Neuroimmune Responses

MSC‐, DPSC‐, and NSC‐derived EV/sEV preparations have been investigated as neuroimmune modulators in selected CNS models [32, 63, 64, 65, 66, 70, 71, 94]. Reported mechanisms include modulation of NLRP3/NF‐κB‐related inflammatory signaling, microglial pyroptosis, autophagy/mitophagy‐linked responses, and regulatory RNA‐mediated effects. These findings are promising but remain context‐dependent; active cargo should be identified as necessary and sufficient rather than inferred from bulk vesicle administration.

### 5.5 Trophic Support, Endogenous Repair, and Limits of Regeneration Claims

EV/sEV‐mediated neural repair should be separated into paracrine support, endogenous repair facilitation, and true structural replacement. Across selected preclinical CNS models, EV/sEV preparations have been associated with neuronal or axonal protection and with inflammatory, vascular, or repair‐related responses [32, 62, 63, 64, 70, 71, 93, 94, 95, 96, 97, 98]. These findings support a paracrine repair‐facilitation model, but they should not be generalized as evidence of de novo neuronal replacement. Evidence for robust, durable neuronal replacement by EV/sEVs is not established; therefore, the most defensible clinical endpoints are target engagement, neuroinflammatory modulation, trophic signaling, barrier stabilization, and functional improvement rather than claims of circuit reconstruction. These EV/sEV‐related mechanisms and delivery modes are summarized in Figure 2.

**FIGURE 2:**
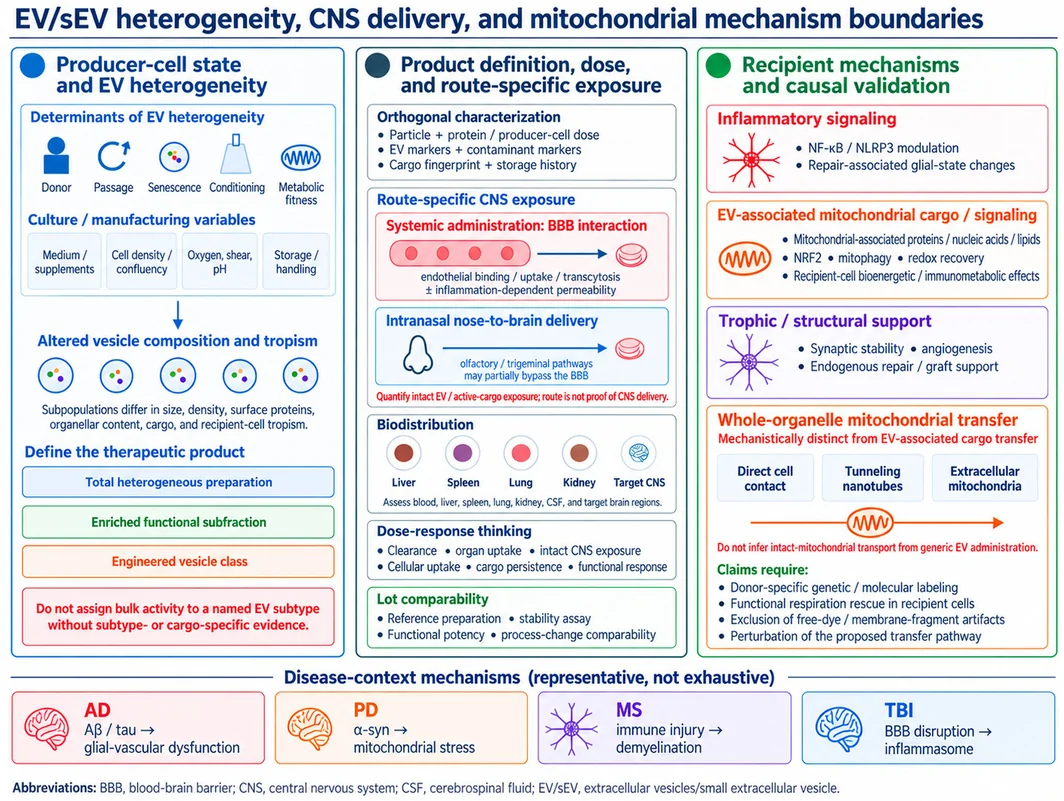
EV/sEV heterogeneity, CNS delivery, and mitochondrial mechanism boundaries. Producer‐cell origin, passage, senescence, conditioning, and metabolic fitness generate overlapping vesicle populations with different size, density, surface composition, organellar content, cargo, and tropism. A translational product should be defined as a total heterogeneous preparation, an enriched functional subfraction, or an engineered vesicle class. Systemic BBB interaction and intranasal nose‐to‐brain delivery are shown separately because olfactory/trigeminal transport may partially bypass the BBB and should not be treated as another BBB‐transcytosis mechanism. Recipient‐cell actions include inflammatory signaling, EV‐associated mitochondrial/metabolic cargo, and trophic/structural support. Whole‐organelle mitochondrial transfer is mechanistically distinct from EV cargo transfer and may involve direct cell contact, tunneling nanotubes, extracellular mitochondria, or other context‐specific routes. Claims that a therapeutic EV/sEV preparation transfers intact mitochondria require donor‐specific labeling, functional respiration rescue, exclusion of labeling artifacts, and perturbation of the proposed transfer pathway. AD, Alzheimer's disease; BBB, blood–brain barrier; CNS, central nervous system; CSF, cerebrospinal fluid; EV, extracellular vesicle; MS, multiple sclerosis; NF‐κB, nuclear factor κB; NLRP3, NLR family pyrin domain containing 3; NRF2, nuclear factor erythroid 2‐related factor 2; PD, Parkinson's disease; PK/PD, pharmacokinetics/pharmacodynamics; sEV, small extracellular vesicle; TBI, traumatic brain injury.

### 5.6 Pharmacokinetics, Pharmacodynamics, Dosing, and Lot Reproducibility

EV/sEVs cannot be treated as a chemically uniform drug class. Apparent exposure is influenced by parent‐cell source, culture state, separation method, co‐isolated proteins or lipoproteins, storage, freeze–thaw history, route of administration, and recipient inflammation. Particle count, total protein, and producer‐cell equivalent are not interchangeable dose units; at least two orthogonal dose descriptors and a mechanism‐linked activity measure should be reported. Brain exposure after intravenous administration should be distinguished from nose‐to‐brain exposure after intranasal administration and quantified rather than inferred from fluorescence alone, with assessment of blood, liver, spleen, lung, kidney, cerebrospinal fluid, and target brain regions because labeling artifacts and uptake by the mononuclear phagocyte system can overestimate intact‐vesicle delivery [52, 53, 54].

A translational PK/PD program should define circulation and clearance, tissue exposure, cellular uptake, persistence of active cargo, repeated‐dose accumulation, dose–response, and the minimum effective dose. Pharmacodynamic endpoints should initially be mechanism‐proximal, such as target RNA or protein modulation, inflammasome suppression, barrier permeability, or glial‐state signatures, before downstream behavioral or clinical outcomes are interpreted. Cargo engineering may improve targeting or potency, but each modification creates new critical quality attributes related to loading efficiency, surface‐ligand density, stability, off‐target distribution, and immunogenicity.

For reproducibility, clinical lots should be bridged using qualified reference preparations, orthogonal particle characterization, contaminant assays, cargo fingerprints, stability‐indicating tests, and validated functional potency assays. Formal comparability studies are required after changes in parent‐cell bank, culture scale, purification, formulation, storage, or analytical platform. MISEV‐aligned reporting is necessary but not sufficient for therapeutic release: it improves transparency and comparability but does not by itself establish product potency, safety, or clinical efficacy.

## 6 Core Mechanisms of Stem Cell and EV/sEV‐Mediated Neuroimmune Niche Reprogramming

The therapeutic effects of stem cell‐based interventions are best interpreted as convergent neuroimmune niche modulation. Rather than treating immunomodulation, trophic support, EV/sEV signaling, metabolic regulation, senescence‐associated remodeling, and selected cell replacement as isolated mechanisms, this framework asks how these mechanisms interact to shift CNS tissue from a hostile disease or injury state toward a repair‐permissive state.

### 6.1 Glial‐State Remodeling and Inflammatory Control

Stem cell products can influence microglia, astrocytes, macrophages, endothelial cells, and T cells through secreted factors, EV/sEVs, and contact‐dependent pathways. CNS‐relevant studies support modulation of NF‐κB/NLRP3‐related inflammatory signaling, MAPK pathways, autophagy/mitophagy‐linked responses, and CD200‐related immune regulation [31, 32, 57, 62, 63, 64, 76, 99, 100, 101]. Future studies should move beyond binary M1/M2 or A1/A2 labels and define glial states with single‐cell, spatial, proteomic, and functional readouts.

### 6.2 Trophic, Synaptic, and Metabolic Support

Secreted trophic factors, EV/sEV‐associated signals, and locally delivered neural or stromal cell products can support synaptic stability, angiogenesis, mitochondrial resilience, and endogenous repair in selected models [19, 20, 34, 35, 70, 71, 72, 73, 74, 75, 76, 77, 78, 79, 80, 81, 82, 94, 95, 102, 103, 104]. This helps explain why transient cell persistence can still produce biological effects. However, trophic improvement does not equal neuronal replacement; integration, connectivity, durability, and functional causality must be directly demonstrated when replacement is claimed.

### 6.3 Mitochondrial Quality Control and Immunometabolic Reprogramming

Mitochondria are active components of the neuroimmune niche rather than downstream markers of injury. Respiratory‐chain dysfunction, excessive reactive oxygen species, altered NAD+/redox balance, calcium stress, and release of mitochondrial DNA or cardiolipin can amplify cGAS‐STING‐ and NLRP3‐related signaling. In parallel, activated microglia, macrophages, astrocytes, and lymphocytes undergo context‐dependent changes in glycolysis, oxidative phosphorylation, fatty‐acid utilization, and amino acid metabolism. An intervention that transiently lowers cytokine concentrations while leaving mitochondrial quality control and maladaptive immunometabolism unchanged may therefore produce only short‐lived niche effects [2, 5, 32, 64].

Stem cells and EV/sEVs may support mitochondrial recovery through NRF2‐linked redox regulation and mitophagy in selected CNS injury models [32, 64]. Other mitochondrial quality‐control domains, including biogenesis and fusion‐fission balance, remain mechanistically relevant but should be demonstrated product‐ and context‐specifically rather than inferred from generic EV administration. Importantly, metabolic rescue mediated by EV‐associated signaling or molecular cargo does not establish transfer of intact mitochondrial organelles.

Intercellular transfer of intact mitochondria has been demonstrated in selected injury systems, including astrocyte‐to‐neuron transfer after stroke and Miro1‐dependent mitochondrial donation by MSCs. These studies provide proof of principle for intercellular organelle rescue but do not demonstrate that therapeutically administered EV/sEV preparations routinely carry intact mitochondria. Whole‐organelle transfer may involve direct cell contact, tunneling nanotubes, extracellular mitochondria or organellar structures, and other context‐specific routes. Product‐specific claims require donor‐specific genetic or molecular labeling, functional respiration measurements, exclusion of free‐dye or membrane‐fragment artifacts, and perturbation of the proposed transfer machinery [105, 106].

The metabolic fitness of the producer cell is also a critical quality attribute. Donor age, passage, senescence, oxygen tension, nutrient conditions, preconditioning, scale‐up, and cryopreservation can alter mitochondrial membrane potential, respiratory reserve, redox state, and EV cargo. Mechanism‐linked potency matrices should therefore consider producer‐cell bioenergetics and recipient‐cell readouts such as mitophagy flux, mitochondrial respiration, oxidative damage, inflammatory‐metabolic state, and restoration of neuronal or glial function [107, 108, 109].

### 6.4 Senescence‐Associated Inflammatory Programs

Senescence‐associated secretory phenotype (SASP) programs contribute to chronic neuroinflammation, glial activation, mitochondrial stress, and impaired stem cell potency [107, 108, 109, 110, 111, 112, 113, 114, 115, 116, 117, 118, 119]. Stem cells and EVs/sEVs may counter selected SASP‐related pathways through SIRT1‐associated resilience, mitochondrial quality control, NF‐κB modulation, and anti‐inflammatory cargo. Current evidence supports effects on senescence‐associated inflammatory signaling and stem cell fitness, but direct senolysis or durable reversal of senescent CNS cell states remains insufficiently established.

### 6.5 Evidence Boundary

Current evidence is strongest for glial immunomodulation, EV/sEV‐mediated cargo transfer, trophic support, and repair‐permissive niche remodeling in preclinical models. Evidence is weaker for durable cell replacement outside selected indications, long‐term circuit reconstruction, direct senescence reversal, and clinically validated nano‐enabled efficacy. These claims should be reserved for studies that include causal mechanism validation, biodistribution, dose–response testing, potency assays, and clinically meaningful biomarkers. Representative clinical evidence for stem cell‐based interventions in selected CNS disorders is summarized in Table 2.

**TABLE 2:** Clinical and regulatory evidence for stem/progenitor cell and EV/sEV interventions in selected CNS disorders.

Study/registry	Product/source/route	Design/*N*/arms	Dose/schedule/follow‐up	Primary endpoint and key findings	Translational interpretation
AD: Lomecel‐B Phase I NCT02600130 [40]	Allogeneic BM‐derived MSC (Lomecel‐B); IV	Randomized, double‐blind, placebo‐controlled; *N* = 33: placebo *n* = 8, 20 M *n* = 15, 100 M *n* = 10	Single infusion; 20 or 100 million cells; follow‐up to 52 weeks	Primary safety endpoint met; no treatment‐related serious adverse events within the prespecified safety period. Cognitive/vascular/biomarker findings exploratory.	Supports early safety and biomarker development; not efficacy proof.
AD: CLEAR‐MIND Phase 2a NCT05233774 [41]	Laromestrocel/Lomecel‐B; IV	Randomized, double‐blind, placebo‐controlled; *N* = 49 across four dosing regimens	Placebo ×4 (*n* = 12); 25 M once then placebo ×3 (*n* = 13); 25 M ×4 (*n* = 13); 100 M ×4 (*n* = 11); monthly; 39 weeks	Primary safety objective met. The secondary composite AD score favored the single 25 M dose followed by placebo ×3 group vs. placebo (difference 0.38, 95% CI −0.06 to 0.82); imaging/atrophy findings were secondary or exploratory.	Safety established in Phase 2a. The nominal composite signal arose from the single‐dose 25 M regimen; dose–response and durability remain uncertain, and efficacy requires confirmation.
AD: intranasal MSC‐exosomes NCT04388982 [51]	Allogeneic adipose MSC‐derived exosome preparation; intranasal	Open‐label, single‐center Phase I/II dose escalation; *N* = 9, three groups (*n* = 3 each)	2 × 10^8^, 4 × 10^8^, or 8 × 10^8^ particles in 1 mL; twice weekly for 12 weeks (24 doses); extended follow‐up	No treatment‐period AEs/SAEs were reported. In the medium‐dose arm, ADAS‐Cog decreased 2.33 (SD 1.19) points at week 12 vs. baseline and 3.98 points through week 36; no significant between‐group amyloid/tau deposition differences were detected.	Direct human EV safety/feasibility evidence, but *N* = 9 and no control arm; cognitive observations are hypothesis‐generating, not confirmatory efficacy evidence.
PD: allogeneic BM‐MSC safety study NCT02611167 [30]	Allogeneic BM‐MSC; IV	Open‐label dose‐escalation; *N* = 20 (*n* = 5 per dose)	Single 1, 3, 6, or 10 × 10^6^ cells/kg infusion; 52 weeks	Primary safety/tolerability objective met; no serious infusion‐related reactions. Motor/inflammatory changes were exploratory.	Established dose‐escalation feasibility; not designed to establish efficacy.
PD: repeated allogeneic BM‐MSC Phase 2 NCT04506073 [42]	Allogeneic BM‐MSC; IV	Randomized, double‐blind, placebo‐controlled; *N* = 45: 3 active *n* = 16; placebo → 2 active *n* = 14; 3 placebo *n* = 15	10 × 10^6^ cells/kg per active infusion; 18‐week intervals; follow‐up 88 weeks	Week‐62 prespecified Bayesian responder endpoint (≥ 5‐point MDS‐UPDRS‐III improvement): 3‐infusion vs. placebo responder‐proportion difference +5.0%, posterior probability 93.7%; 2‐infusion vs. placebo −62.4%, posterior probability ≥ 99.9%. Mean MDS‐UPDRS‐III improvements were 16.9, 3.9, and 14.6 points in the 3‐infusion, 2‐infusion, and placebo arms, respectively.	Marked schedule discordance prevents uncomplicated efficacy inference and highlights dose/schedule uncertainty, functional potency, batch variability, and lot comparability.
PD: iPSC‐derived dopaminergic progenitors jRCT2090220384/UMIN000033564 [36]	Allogeneic iPSC‐derived dopaminergic progenitors; bilateral putamen	Phase I/II, open‐label; *N* = 7	2.4 × 10^6^ cells/putamen in the low‐dose cohort (first 3 participants) or 4.2–5.4 × 10^6^ cells/putamen in the high‐dose cohort; bilateral stereotactic grafting; 24‐month primary follow‐up.	Primary safety/engraftment objectives met; no tumor/outgrowth. Safety *N* = 7; efficacy analysis *N* = 6. Putaminal 18F‐DOPA Ki increased 44.7%; motor changes were exploratory.	Strong mechanism‐endpoint fit for cell replacement; small uncontrolled trial limits comparative efficacy inference.
PD: hESC‐derived dopaminergic progenitors NCT04802733 [37]	Bemdaneprocel, hESC‐derived dopaminergic progenitors; bilateral putamen	Open‐label Phase I; *N* = 12: low dose *n* = 5, high dose *n* = 7	0.9 M or 2.7 M cells per putamen; 1‐year immunosuppression; ≥ 18 months reported follow‐up	Primary safety/tolerability objectives met; no product‐related serious safety signal. At 18 months, FDOPA uptake increased and the high‐dose cohort showed a mean 23‐point MDS‐UPDRS‐III OFF improvement; these PET and motor findings were secondary/exploratory.	Supports continued definitive testing and manufacturing standardization.
MS: MESEMS NCT01854957 [43]	Autologous BM‐MSC; IV	Phase 2 randomized, double‐blind crossover; *N* = 144: early MSC *n* = 69, placebo‐first *n* = 75	Single 1–2 × 10^6^ cells/kg infusion; crossover at 24 weeks; ~48‐week efficacy follow‐up	Primary efficacy endpoint (Gd‐enhancing lesions) not met: rate ratio 0.94 (95% CI 0.58–1.50), *p* = 0.78; safety acceptable.	Neutral efficacy despite biological plausibility; underscores need for exposure, potency, patient selection and sensitive endpoints.
MS: human fetal neural progenitors NCT03269071 [33]	Human fetal neural progenitor cells; intrathecal	Open‐label Phase I; *N* = 12	0.7, 1.4, 2.8, or 5.7 × 10^6^ viable cells/kg; single intrathecal dose; 2 years	Primary safety/tolerability objective met; no severe product‐related adverse events; biomarker/atrophy signals exploratory.	Supports feasibility/neuroprotective hypothesis, not efficacy.
MS: MSC‐neural progenitors NCT03355365 [120]	Autologous MSC‐NP; intrathecal	Phase II randomized, double‐blind, placebo‐controlled; *N* = 54: 27 vs. 27	Six intrathecal injections every 2 months; active vs. saline	Primary EDSS Plus endpoint not improved: 33% vs. 37%; walking/bladder/MRI/CSF subgroup findings exploratory.	Neutral primary outcome; subgroup observations require prospective confirmation.
ALS: MSC‐NTF/NurOwn NCT03280056 [44]	Autologous MSCs induced to secrete neurotrophic factors; intrathecal	Randomized placebo‐controlled Phase 3; *N* = 189: active 95, placebo 94	100–125 × 10^6^ cells; three intrathecal injections every 8 weeks; 28‐week primary assessment	Primary responder endpoint not met: 33% vs. 28%; OR 1.33, *p* = 0.45. Secondary efficacy endpoints also not met.	Biomarker and biological rationale did not translate into prespecified clinical benefit.
ALS: spinal cord‐derived NSC NCT01348451 [45]	Human spinal cord‐derived NSCs; intraspinal	Phase I; primary report *N* = 15 undergoing 18 surgeries	Dose‐escalation, up to 1.5 M cells depending on cohort; cervical/lumbar delivery	Primary safety/feasibility objective: procedure did not accelerate disease progression; no efficacy claim.	Delivery feasibility established; efficacy and optimal dose remain unresolved.
TBI: pediatric BMMNC NCT01851083 [49]	Autologous heterogeneous BMMNC fraction; IV within 48 h	Randomized, double‐blind, placebo‐sham controlled; *N* = 47; 3:2 allocation	6 or 10 × 10^6^ cells/kg vs. placebo; 1 month, 6 months, and 1 y assessments	Primary MRI endpoints assessed quantitative brain volumes and corpus‐callosum microstructural integrity at 6 and 12 months. At 1 year, white‐matter volume loss was lower with BMMNC vs. placebo (mean difference −20,600 mm^3^, 95% CI −35,868 to −5332; *p* = 0.01); no clear functional‐group difference was demonstrated.	Objective imaging signal in acute injury; BMMNCs are heterogeneous fractions, not purified stem cells.
TBI: adult BMMNC NCT01575470 [50]	Autologous heterogeneous BMMNC fraction; IV within 48 h	Dose‐escalation clinical study; *N* = 25: 15 treated, 10 controls	6, 9, or 12 × 10^6^ cells/kg; imaging follow‐up at ~1 and 6 months	Primary objective was safety/logistical feasibility: no serious harvest‐ or infusion‐related adverse events; mild clinically insignificant pulmonary toxicity occurred at 12 × 10^6^ cells/kg. Imaging/inflammatory findings were exploratory.	Supports feasibility and time‐window optimization; efficacy remains unresolved.

*Note:* FTD and HD are not listed separately because disease‐specific therapeutic clinical evidence remains predominantly preclinical. The table now includes direct human EV evidence and explicitly distinguishes heterogeneous BMMNC fractions from purified stem‐cell products. *N* denotes participants in the primary report; where a requested study parameter was not reported, no value was inferred. Regulatory authorization of AMCHEPRY/raguneprocel is discussed in Sections 8.2 and 7.7 because it is a posttrial regulatory status rather than an additional clinical trial row.

Abbreviations: AD, Alzheimer's disease; ALS, amyotrophic lateral sclerosis; BMMNC, bone marrow mononuclear cell; BM‐MSC, bone marrow mesenchymal stromal cell; EDSS, Expanded Disability Status Scale; EV, extracellular vesicle; hESC, human embryonic stem cell; iPSC, induced pluripotent stem cell; IV, intravenous; MSC‐NP, mesenchymal stromal cell‐neural progenitor; PD, Parkinson's disease; TBI, traumatic brain injury.

Applying the operational criteria at study level (Table S1) shows that most current interventions support transient immunomodulation or partial/probable niche remodeling (Levels 1–2). Coordinated multi‐compartment reprogramming and especially causal, durable reprogramming (Levels 3–4) remain uncommon because cross‐compartment causality and persistence are rarely demonstrated together. Clinical status alone does not increase this mechanistic evidence level.

### 6.6 Determinants of Successful and Failed Translation

The literature does not support a simple conclusion that stronger immunosuppression, a higher cell dose, or more complex engineering necessarily produces better clinical outcomes. Apparent success is more likely when the dominant pathology is biologically accessible to the product, intervention occurs within a responsive disease window, exposure at the target tissue is demonstrated, lot potency predicts target engagement, and the clinical endpoint reflects the proposed mechanism. Acute or subacute injury and inflammation‐dominant states may be more responsive to transient paracrine products than chronic diffuse neurodegeneration, whereas focal cell loss such as nigrostriatal denervation offers a more coherent indication for lineage‐specific replacement.

Conversely, promising animal studies frequently overestimate translation because models are young, genetically or environmentally homogeneous, treated early, and assessed over short intervals. Small sample sizes, incomplete blinding, selective reporting, species‐specific immune biology, supraclinical dosing, and outcome measures that do not map to patient disability can further inflate apparent efficacy. In clinical trials, disease‐stage heterogeneity, insufficient exposure, variable manufacturing, poorly validated potency assays, and reliance on exploratory subgroup or biomarker signals can obscure or exaggerate benefit.

Negative and neutral trials should therefore be treated as mechanistic experiments rather than publication failures. A neutral primary endpoint may indicate an ineffective product, but it may also reveal a mismatch among mechanism, patient selection, dose, route, timing, or endpoint. Progression to larger trials should require independent replication, prespecified dose–response and biodistribution data, lot‐to‐lot potency comparability, a target‐engagement biomarker, and a clinically interpretable go/no‐go threshold [43, 44, 120].

The clinical evidence illustrates the distinction between feasibility and efficacy. The Phase I/II intranasal adipose MSC‐exosome study in AD provides direct human EV safety/feasibility evidence but remains too small and uncontrolled for efficacy conclusions [51]. In PD, the 2025 repeated allogeneic BM‐MSC Phase 2 study produced discordant treatment‐arm findings, reinforcing the importance of functional potency and batch comparability rather than providing simple efficacy confirmation [42]. MESEMS and the MSC‐NP study in MS and NurOwn in ALS did not meet their prespecified primary efficacy endpoints [43, 44, 120]. By contrast, iPSC‐ and hESC‐derived dopaminergic progenitor trials in PD have a tighter replacement mechanism–endpoint relationship, but their small open‐label designs primarily establish safety, feasibility, and target engagement [36, 37]. The March 2026 conditional and time‐limited Japanese authorization of AMCHEPRY/raguneprocel represents an important regulatory milestone, but post‐marketing confirmation is required and should not be equated with definitive long‐term comparative efficacy [46, 47].

## 7 Enabling Technologies: Engineered EV/sEV Delivery, Nanocarriers, Biomaterials, and Tracking

### 7.1 Engineered EV/sEV and Nanocarrier Platforms

Nanocarriers and engineered EV/sEVs can improve targeted delivery, cargo protection, retention, controlled release, and BBB interaction. In CNS disorders, their role should be defined pragmatically: they may improve homing, local bioavailability, target engagement, and pharmacological interpretability, but they do not eliminate product heterogeneity or prove clinical efficacy by themselves [27, 28, 29, 53].

Hybrid EV‐liposome systems, biomimetic nanocarriers, CCR2‐enriched EV‐coated formulations, TAT‐functionalized lipid nanoparticles, and exosome‐curcumin or antisense oligonucleotide systems illustrate how engineered platforms can coordinate anti‐inflammatory, antioxidant, or aggregate‐modulating effects [27, 28, 29, 121, 122, 123, 124, 125, 126, 127, 128, 129, 130, 131, 132, 133]. Each added engineering step also creates new variables in yield, purification, toxicity, off‐target delivery, biodistribution, and regulatory classification.

### 7.2 Biomaterials and Local Retention

Hydrogels, decellularized extracellular‐matrix bioinks, conductive graphene or black‐phosphorus materials, scaffolds, and cell‐attached or electroactive micropatches can improve local retention and provide biochemical, structural, or electrical cues for stem‐cell survival, differentiation, and tissue repair [35, 81, 82, 102, 103, 104, 128, 129, 133, 134, 135, 136]. Their clinical value should be judged by whether they improve target engagement, survival, and functional endpoints compared with cells or EV/sEVs alone.

### 7.3 Cell Tracking and Biodistribution

Tracking platforms are essential because clinical outcomes cannot be interpreted mechanistically unless cell or EV/sEV distribution, persistence, and clearance are measured. Multimodal cell‐tracking approaches include fluorescence, PET, photoacoustic imaging, and MRI, whereas EV biodistribution studies commonly use optical, nuclear, and magnetic‐resonance‐based labeling strategies [137, 138, 139]. However, labels and tracers can dissociate from the administered product, persist after product clearance, or alter viability and cargo behavior; these artifacts must therefore be evaluated before imaging signal is interpreted as intact‐cell or intact‐vesicle exposure.

### 7.4 Comparative Selection of Engineered Delivery Platforms

No engineered platform is universally superior. Native EV/sEVs permit cell‐free and potentially repeatable dosing but often show rapid systemic clearance and uncertain intact‐vesicle CNS exposure. Surface‐ or cargo‐engineered EVs may improve targeting and mechanism‐specific potency, yet additional modification and purification can lower yield and create new attributes that must be controlled. Synthetic liposomes and nanoparticles can offer more defined composition and scalable cargo loading, but they may lack endogenous tropism and introduce material‐specific toxicity or innate immune activation. Hydrogels and scaffolds improve local retention and structural support for focal lesions or grafts, whereas cell‐attached micropatches and electroactive materials add spatial or electrical cues at the cost of invasive delivery and combination‐product complexity [27, 28, 29, 34, 35, 81, 82, 93, 102, 104, 125, 130, 131, 132, 133, 134, 135].

Comparative development should use the least‐complex adequate platform as the reference. A more complex system should advance only when it provides a prespecified improvement in target‐region exposure, retention, duration, dose reduction, or functional benefit without disproportionate toxicity, manufacturing variability, or regulatory burden. Head‐to‐head biodistribution, potency, stability, and safety studies are therefore more informative than comparison with an untreated control alone.

### 7.5 Potency Assays, Biomarkers, GMP, and Regulatory Pathways

For clinical translation, potency assays and release criteria should be defined early and linked to the intended mechanism of action. Candidate products should be linked to specific mechanisms such as NLRP3 suppression, glial‐state remodeling, trophic factor secretion, mitochondrial stabilization, barrier repair, or EV/sEV cargo delivery. Release assays should include identity, viability, purity, sterility, genomic safety when relevant, EV/sEV markers and contaminants, active‐cargo quantification, and functional potency tests that predict target engagement.

Potency should not be reduced to a single surface marker, cytokine concentration, differentiation marker, or particle count. A fit‐for‐purpose potency matrix should combine identity, purity, and viability with one or more validated functional assays that are proximal to the claimed mechanism of action. For immunomodulatory MSCs, examples include suppression of activated immune‐cell outputs or glial inflammasome activity; for neural progenitors, survival, differentiation fidelity, and trophic function; and for EV/sEV products, delivery of active cargo and a recipient‐cell functional response. Assays used for lot release should be rapid and robust, whereas deeper characterization assays can support process development and comparability. Regulatory guidance for cell‐based medicinal products similarly emphasizes early development of potency assays linked to intended biological activity and product‐specific release specifications [48]. When mitochondrial or immunometabolic rescue is part of the proposed mechanism, potency testing should also evaluate producer‐cell respiratory fitness and a recipient‐cell response such as mitophagy flux, oxidative phosphorylation, mitochondrial danger signaling, or reversal of an inflammatory metabolic program.

Reproducibility also depends on controlling donor age and comorbidity, tissue source, passage number, senescence, culture media, oxygen tension, preconditioning, scale‐up platform, cryopreservation, thaw‐to‐use interval, and operator or site effects. Critical quality attributes and acceptance ranges should be prospectively defined, with qualified reference lots, stability‐indicating assays, and formal comparability studies after any process change. For EV/sEVs, release testing should additionally address parent‐cell identity, particle and protein metrics, size distribution, EV‐enriched and contaminant markers, residual process reagents, sterility and endotoxin, cargo fingerprint, storage, freeze–thaw sensitivity, and mechanism‐linked bioactivity. Interlaboratory transfer and ring‐testing are needed because internal consistency within one laboratory does not establish external reproducibility.

For GMP translation, chemistry, manufacturing, and controls (CMC) planning should address donor variability, cell banking, culture conditions, EV/sEV isolation, cryopreservation, scalability, sterility, release criteria, product stability, batch comparability, and cost. Regulatory classification is especially complex for combination products that integrate cells, EV/sEVs, nanocarriers, biomaterials, gene editing, or devices. Early interaction with regulatory agencies and predefined long‐term safety surveillance are therefore essential development tasks rather than late‐stage administrative details. The main enabling technologies and translational bottlenecks are summarized in Figure 3.

**FIGURE 3:**
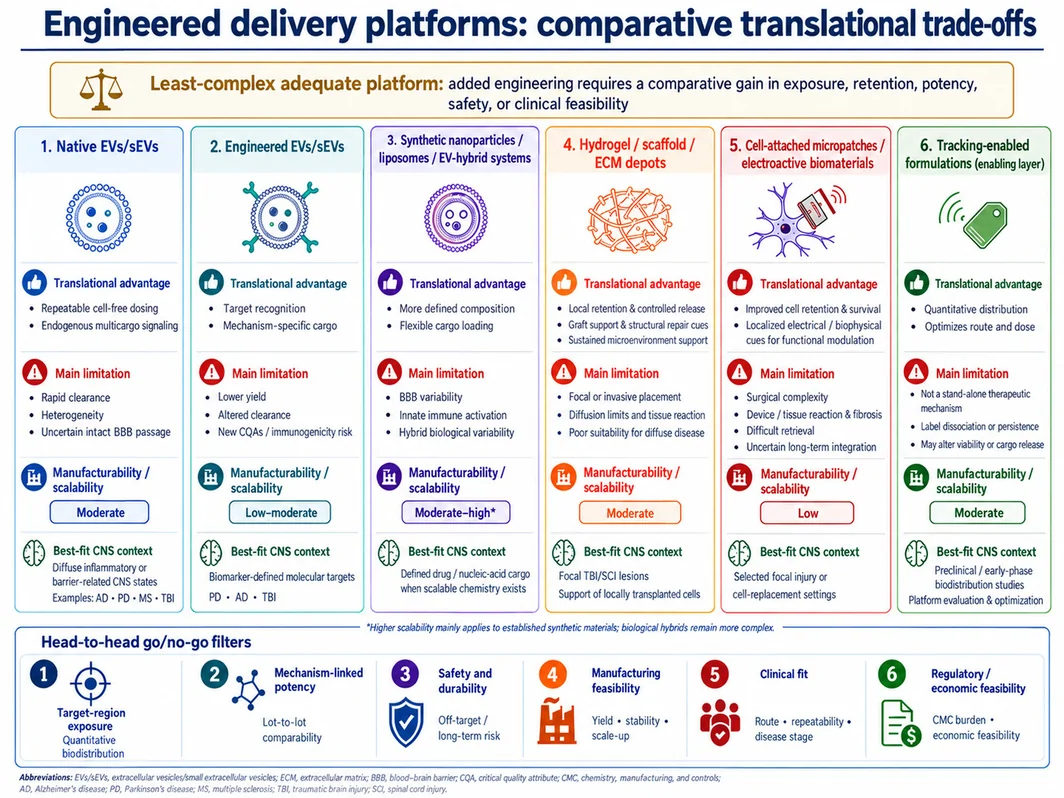
Engineered delivery platforms: comparative translational trade‐offs. Native EVs/sEVs, engineered EVs/sEVs, synthetic nanoparticle or liposome systems, EV‐hybrid formulations, hydrogels or scaffolds, extracellular‐matrix depots, cell‐attached micropatches, electroactive biomaterials, and tracking‐enabled formulations differ in repeatability, target recognition, CNS exposure, local retention, invasiveness, manufacturability, scalability, and chemistry, manufacturing, and controls burden. The least‐complex adequate platform should serve as the reference: added engineering should advance only when head‐to‐head studies demonstrate a prespecified gain in target‐region exposure, retention, mechanism‐linked potency, safety, or clinical feasibility. Go/no‐go assessment should integrate quantitative biodistribution, lot‐to‐lot potency comparability, durability and off‐target risk, manufacturing feasibility, disease‐stage and route fit, and a credible regulatory and economic pathway. AD, Alzheimer's disease; BBB, blood–brain barrier; CMC, chemistry, manufacturing, and controls; CNS, central nervous system; CQA, critical quality attribute; ECM, extracellular matrix; EVs, extracellular vesicles; MS, multiple sclerosis; PD, Parkinson's disease; SCI, spinal cord injury; sEVs, small extracellular vesicles; TBI, traumatic brain injury.

### 7.6 Ethical and Long‐Term Safety Considerations

The ethical profile differs substantially among platforms. For embryonic or fetal tissue‐derived products, provenance, donor consent, traceability, and jurisdiction‐specific restrictions require explicit documentation. iPSC‐derived products reduce some source‐related concerns but introduce others, including genomic privacy, reprogramming‐associated abnormalities, clonal selection, residual pluripotent cells, and the possibility of ectopic tissue or tumor formation. Because intracranial grafts may persist for years and cannot be readily retrieved, consent should address irreversible implantation, uncertain durability, immunosuppression, graft‐related adverse effects, and the need for prolonged imaging and clinical surveillance.

Long‐term follow‐up should be risk‐based and include predefined monitoring for neoplasia, ectopic growth, infection, alloimmunity, loss of efficacy, and procedure‐related complications. Donor‐product‐recipient traceability, registries, and harmonized adverse‐event reporting are particularly important for allogeneic and banked products. EV/sEV products avoid uncontrolled cellular proliferation but are not intrinsically risk‐free; engineered or poorly characterized preparations may distribute to off‐target organs, transfer pro‐inflammatory, procoagulant, oncogenic, or immunogenic cargo, and create cumulative risks with repeated dosing.

Ethical translation also requires protection against therapeutic misconception and unregulated commercial use. The vulnerability of patients with progressive neurological disease increases the importance of transparent communication about the distinction among experimental safety, target engagement, and proven efficacy. Equitable access, donor compensation, ownership of cell lines and derived products, and the financial burden of long‐term monitoring should be considered early rather than after efficacy claims have been made. Regulatory authorities emphasize manufacturing consistency, tumorigenicity assessment, traceability, pharmacovigilance, and long‐term surveillance for advanced cell‐based products [48, 140, 141].

### 7.7 Regulatory, Commercialization, and Health‐Economic Readiness

Regulatory classification is jurisdiction‐ and product‐dependent. In the United States, the degree of manipulation and homologous use influence whether a human cell or tissue product can follow the HCT/P pathway or requires full biologics regulation; in the European Union, substantially manipulated or non‐homologously used cell products are generally evaluated as advanced therapy medicinal products, and cell‐device or cell‐biomaterial combinations may be classified as combined ATMPs [48, 140, 141]. Engineered EV/sEV products, cargo‐loaded vesicles, and cell‐EV‐device combinations may follow different or still‐evolving pathways, making early scientific advice essential. In the United States, no exosome products are currently FDA approved; CNS EV/sEV products therefore remain investigational unless otherwise specified [55].

Commercial readiness requires a reproducible supply chain as well as biological efficacy. Developers must decide between centralized and point‐of‐care manufacturing, establish chain of identity and custody, validate cryoshipping and storage, qualify treatment centers, and show that process scale‐up does not alter potency. Intracranial delivery, immunosuppression, repeat dosing, imaging, and long‐term surveillance add resource use that is rarely captured in early trials. Formal CNS‐specific cost‐effectiveness evidence for these platforms remains sparse [56]; reimbursement and economic sustainability should therefore be framed as prospective development considerations rather than established empirical conclusions. Durable functional benefit, reduction in downstream care, and comparison with existing therapies will ultimately determine value.

International harmonization of nomenclature, release criteria, reference standards, adverse‐event definitions, and posttreatment registries would reduce duplicative development. For academic products, regulatory support, intellectual‐property planning, and technology transfer should begin before pivotal manufacturing decisions, because late conversion from exploratory laboratory methods to a controlled commercial process can invalidate comparability and delay patient access.

## 8 Disease‐Specific Therapeutic Logic and Evidence Boundaries in Selected CNS Disorders

TBI is included as an acute/subacute CNS injury context that informs neuroimmune niche modulation, whereas AD, PD, FTD, ALS, MS, and HD illustrate disease‐specific boundaries across chronic neurodegenerative and immune‐mediated conditions. Stroke, spinal cord injury, subarachnoid hemorrhage, and optic nerve injury are used selectively for cross‐cutting mechanistic or delivery evidence rather than treated as equivalent disease‐specific therapeutic sections.

### 8.1 Alzheimer's Disease

AD involves Aβ pathology, tau pathology, synaptic failure, vascular dysfunction, chronic glial activation, and aging‐associated inflammatory programs. Stem cell‐based strategies should be framed primarily around immunomodulation, trophic support, BBB or vascular‐neuroimmune communication, and synaptic preservation rather than large‐scale neuronal replacement [96, 97, 142, 143, 144, 145, 146, 147]. Clinical studies of Lomecel‐B/laromestrocel provide early safety and biomarker‐oriented evidence, but larger efficacy‐powered trials with amyloid/tau, neuroinflammation, vascular, cognitive, and digital biomarkers are needed [40, 41].

### 8.2 Parkinson's Disease

PD requires a stage‐adapted framework. In earlier disease stages, MSC‐derived products, EV/sEVs, and engineered delivery may target neuroinflammation, mitochondrial stress, alpha‐synuclein‐associated toxicity, and trophic support [148, 149, 150, 151, 152, 153, 154, 155, 156, 157, 158, 159]. A 2025 randomized Phase 2 trial of repeated intravenous allogeneic BM‐MSCs in 45 participants produced discordant findings across infusion schedules: the three‐infusion arm crossed the prespecified Bayesian probability threshold relative to placebo, whereas the two‐infusion arm performed substantially worse than placebo. Rather than establishing uncomplicated efficacy, this pattern highlights the need for mechanism‐linked functional potency assays, lot‐to‐lot comparability, and prospective dose‐schedule validation [42]. In advanced dopaminergic denervation, iPSC‐ or hESC‐derived dopaminergic progenitors provide a more coherent replacement strategy, with early trials reporting safety, graft survival/dopamine‐production signals, and exploratory clinical or imaging outcomes [36, 37]. In March 2026, AMCHEPRY (raguneprocel), an allogeneic iPSC‐derived dopaminergic neural progenitor product, received conditional and time‐limited manufacturing and marketing authorization in Japan; post‐marketing clinical confirmation and surveillance are required before full approval [46, 47]. Replacement and niche modulation remain complementary because an inflamed or metabolically stressed host environment may compromise graft survival and function.

### 8.3 Traumatic Brain Injury

TBI provides a clinically relevant model of acute/subacute neuroimmune niche disruption. MSCs, NSCs, heterogeneous bone marrow mononuclear cell (BMMNC) fractions, and EV/sEV products have mainly been tested to limit secondary injury, BBB disruption, edema, microglial activation, inflammasome signaling, and apoptosis while supporting angiogenesis and endogenous repair [49, 50, 93, 98, 134, 135, 136, 160, 161, 162, 163, 164, 165, 166, 167, 168]. BMMNC preparations contain multiple hematopoietic and progenitor populations and should not be interpreted as purified stem‐cell products. The strongest rationale is early modulation of secondary injury cascades; translation requires precise treatment windows, route comparisons, biodistribution, and objective neuroimaging or functional endpoints.

### 8.4 Frontotemporal Dementia

FTD is genetically and pathologically heterogeneous. GRN haploinsufficiency, C9orf72 repeat expansion, MAPT pathology, TDP‐43 aggregation, lysosomal dysfunction, RNA dysregulation, and proteostasis failure make generic cell therapy unlikely to be sufficient [169, 170, 171, 172, 173, 174, 175, 176, 177, 178]. iPSC‐derived neurons, glia, and organoids are particularly valuable for patient‐specific mechanism testing and therapeutic screening. Stem cell‐derived therapeutic products remain exploratory and should be aligned with genetic subtype and evidence of proteostasis or lysosomal rescue.

### 8.5 Amyotrophic Lateral Sclerosis

ALS illustrates the challenge of treating diffuse network and motor neuron degeneration. MSC‐NTF/NurOwn trials showed that intrathecal trophic cell products can be delivered safely, but the Phase 3 study did not meet primary or secondary efficacy endpoints [44]. Intraspinal NSC transplantation established procedural feasibility in early trials [45]. Future ALS approaches should connect potency assays, neurotrophic secretion, anti‐inflammatory activity, CSF biomarkers, and patient selection to clinically meaningful progression endpoints.

### 8.6 Multiple Sclerosis

MS differs from classical neurodegenerative disorders because immune‐mediated demyelination, compartmentalized inflammation, and progressive neurodegeneration interact. Intravenous MSC therapy in MESEMS demonstrated safety but limited activity [43], while intrathecal neural progenitor and MSC‐neural progenitor approaches reported feasibility and exploratory neuroprotective or functional signals [33, 120]. Translation should focus on progressive MS phenotypes, remyelination, axonal preservation, CSF biomarkers, MRI atrophy metrics, and careful separation from hematopoietic stem cell transplantation paradigms.

### 8.7 Huntington's Disease

HD is a monogenic striatal neurodegenerative disorder driven by mutant huntingtin, medium spiny neuron vulnerability, mitochondrial stress, synaptic dysfunction, and neuroimmune changes. Stem cell strategies have mainly focused on iPSC disease modeling, medium spiny neuron replacement, trophic support, and genome‐editing‐compatible platforms [158, 179]. Compared with PD, clinical replacement evidence remains less mature; therefore, HD should be discussed as a mechanistically plausible but still early translational frontier.

### 8.8 Cross‐Disease Mechanism and Delivery Matching

Across disorders, translation appears most plausible when product action is anatomically and temporally matched to disease biology. Focal, well‐defined cell loss favors replacement; acute or subacute inflammatory injury favors time‐limited paracrine modulation; and chronic diffuse neurodegeneration requires repeatable, biomarker‐guided interventions that can reach multiple regions without unacceptable cumulative risk. No single product is therefore expected to dominate across AD, PD, ALS, MS, FTD, HD, and TBI. A clinically useful framework should first classify the patient by dominant niche state: active inflammation, barrier dysfunction, trophic insufficiency, demyelination, or replacement‐relevant cell loss, and then select the least complex product capable of achieving measurable target engagement. Delivery‐platform choice should then be based on the required anatomical reach, duration, repeatability, invasiveness, and manufacturing feasibility rather than on technological novelty alone. Study‐level application of the four operational niche criteria is summarized in Table S1; most current interventions are classified as transient immunomodulation or partial/probable remodeling rather than causal and durable multi‐compartment reprogramming.

## 9 A Translational Roadmap: Go/No‐Go Criteria for Clinical Progression

### 9.1 Mechanistic and Preclinical Readiness

Before first‐in‐human testing, the intended mechanism of action should be experimentally distinguishable from nonspecific trophic or immunosuppressive effects. Evidence should include causal perturbation, dose–response, clinically relevant timing, biodistribution, and durability in at least one model that reflects age, disease stage, and host immunity. Independent replication or multicenter preclinical confirmation is especially valuable for products with complex manufacturing.

### 9.2 Product and Manufacturing Readiness

Each clinical lot should meet predefined identity, purity, viability or particle‐characteristic, sterility, stability, and functional potency specifications. Lot release should use validated assays with acceptance ranges linked to target engagement, and process changes should trigger comparability studies. Failure to demonstrate this linkage should be a no‐go criterion even when exploratory animal efficacy is positive.

### 9.3 Clinical and Regulatory Readiness

Early trials should predefine whether their objective is safety, target engagement, dose finding, or efficacy and should avoid interpreting secondary or post hoc signals as confirmatory. Patient selection, route, treatment window, immunosuppression, repeat dosing, and endpoints should be matched to the mechanism of action. Trials should incorporate exposure and target‐engagement biomarkers, prospective stopping rules, long‐term surveillance, and transparent reporting of neutral and harmful outcomes.

### 9.4 Decision Principle

A practical development decision should require six aligned elements: a disease‐stage‐specific biological rationale; a sufficiently characterized product; reproducible potency across lots; demonstrated target‐tissue exposure; a prespecified biomarker of target engagement; and a clinically meaningful endpoint with a feasible regulatory and economic pathway. Absence of any one element should prompt redesign rather than escalation to a larger trial. This framework provides criteria by which proposed niche‐modulating interventions can be experimentally evaluated, rather than a broad label applied retrospectively to heterogeneous interventions. These prospective go/no‐go criteria complement the retrospective study‐level evidence calibration presented in Table S1.

## 10 Conclusion and Future Perspectives: Mechanism‐Defined Neuroimmune Niche Reprogramming

Stem cell and EV/sEV strategies for selected CNS disorders are moving from broad regenerative claims toward mechanism‐defined neuroimmune niche modulation. The strongest current evidence supports paracrine immunomodulation, EV/sEV‐mediated cargo transfer, trophic and synaptic support, barrier communication, and partial restoration of repair‐permissive tissue states. Durable neuronal replacement is most plausible in selected focal‐loss disorders such as PD, whereas diffuse disorders require disease‐specific, mechanism‐linked strategies. The framework should be applied conditionally: it does not imply that all pathological niches are reversible or that multi‐mechanistic products are inherently superior to simpler, well‐characterized interventions. Accordingly, isolated cytokine suppression should be described as immunomodulation, whereas neuroimmune niche reprogramming should be reserved for coordinated and sufficiently durable changes across interacting tissue compartments that are linked to target engagement and functional outcome. Table S1 provides study‐level calibration of these evidence boundaries.

### 10.1 Defined Biologics and Potency

Future products should be treated as defined biologics with measurable identity, purity, cargo composition, and functional potency. Potency assays should map directly to intended mechanisms such as inflammasome inhibition, glial‐state remodeling, trophic support, mitochondrial stabilization, BBB repair, or target‐cell cargo delivery. For EV/sEV products, MISEV2023‐aligned characterization should be considered the minimum reporting standard [53].

### 10.2 Biomarkers, Patient Selection, and Target Engagement

Clinical development should shift from generic functional outcomes to biomarker‐linked evidence of target engagement. Imaging, fluid biomarkers, digital measures, inflammatory signatures, and disease‐stage stratification can help identify patients with inflammation‐dominant, synaptic failure‐dominant, demyelinating, replacement‐relevant, or acute‐injury niches. These tools must be prospectively validated before they guide therapy selection.

### 10.3 Manufacturing and Regulatory Readiness

The field must address donor variability, cell banking, culture conditions, EV/sEV isolation, cryopreservation, scalability, sterility, release criteria, product stability, and cost. Combination products involving engineered EV/sEVs, nanocarriers, biomaterials, devices, or gene modification require early regulatory planning and long‐term safety surveillance.

Overall, stem cell‐based therapy should be developed as a mechanism‐defined platform for neuroimmune niche modulation rather than as a universal regenerative cure. This framework reduces overstatement, clarifies testable mechanisms, and provides a stronger basis for translating cell‐based and cell‐free interventions into clinically reliable CNS therapeutics. Future progress will depend as much on learning from neutral trials and manufacturing failures as on reporting positive preclinical findings. The most credible candidates will be those that can be reproduced across laboratories and lots, show quantitative CNS exposure and target engagement, and deliver durable patient benefit at an acceptable procedural, regulatory, and economic burden.

## Author Contributions

**Wenjing Lu:** writing – original draft and literature review. **Junqiang Yan:** critical revision and intellectual content revision. **Hongfang Feng:** conceptualization, supervision, manuscript revision, and final approval.

## Funding

This work was funded by the Science and Technology Department of Henan Province (Henan Provincial Department of Science and Technology), No. 242103810041 (to J.Y.).

## Ethics Statement

Ethical approval was not required because this article is a narrative review of previously published literature.

## Conflicts of Interest

The authors declare no conflicts of interest.

## Supporting information


**Table S1:** Study‐level application of the neuroimmune niche reprogramming framework.
**Data S1:** Supplementary methods. Literature search and clinical‐evidence selection.

## Data Availability

Data sharing is not applicable to this article as no datasets were generated or analyzed during this study.
