# Supplementary material for: Stem Cell and EV/sEV Strategies for Neuroimmune Niche Reprogramming in Selected CNS Disorders: Mechanisms, Engineered Delivery, and Translational Readiness
Source: CNS Neurosci Ther. 2026 Oct 21;32(10):e71211. doi: 10.1002/cns.71211 (PMC13685674; doi:10.1002/cns.71211)
Supplement: Supplementary file 1 [file CNS-32-e71211-s001.docx]

Supplementary Materials

**Manuscript ID: 9314844**

*Stem Cell and EV/sEV Strategies for Neuroimmune Niche Reprogramming in Selected CNS Disorders*

# Supplementary Methods. Literature search and clinical-evidence selection

Search coverage: the final narrative-review search window covered January 1, 2010 through June 30, 2026. PubMed, Web of Science Core Collection, Scopus, and ClinicalTrials.gov were included. June 30, 2026 was used as the final literature cutoff for the second revision. Official regulatory webpages were consulted separately to verify authorization status; webpage access dates are reported in the main reference list and do not alter the literature cutoff. The review is narrative; no quantitative meta-analysis was performed.

## PubMed

(("stem cell"[Title/Abstract] OR "mesenchymal stromal cell*"[Title/Abstract] OR "mesenchymal stem cell*"[Title/Abstract] OR "neural stem cell*"[Title/Abstract] OR "neural progenitor cell*"[Title/Abstract] OR "induced pluripotent stem cell*"[Title/Abstract] OR "embryonic stem cell*"[Title/Abstract] OR "dental pulp stem cell*"[Title/Abstract] OR "extracellular vesicle*"[Title/Abstract] OR "small extracellular vesicle*"[Title/Abstract] OR exosome*[Title/Abstract]) AND ("central nervous system"[Title/Abstract] OR brain[Title/Abstract] OR Alzheimer*[Title/Abstract] OR Parkinson*[Title/Abstract] OR "amyotrophic lateral sclerosis"[Title/Abstract] OR "multiple sclerosis"[Title/Abstract] OR Huntington*[Title/Abstract] OR "frontotemporal dementia"[Title/Abstract] OR "traumatic brain injury"[Title/Abstract] OR "ischemic stroke"[Title/Abstract] OR "spinal cord injury"[Title/Abstract] OR "subarachnoid hemorrhage"[Title/Abstract] OR "optic nerve injury"[Title/Abstract]) AND (neuroimmune[Title/Abstract] OR microglia*[Title/Abstract] OR astrocyte*[Title/Abstract] OR inflammasome[Title/Abstract] OR NLRP3[Title/Abstract] OR "blood-brain barrier"[Title/Abstract] OR senescence[Title/Abstract] OR biodistribution[Title/Abstract] OR pharmacokinetic*[Title/Abstract] OR potency[Title/Abstract] OR biomarker*[Title/Abstract] OR GMP[Title/Abstract] OR nanocarrier*[Title/Abstract] OR biomaterial*[Title/Abstract] OR "clinical trial"[Title/Abstract])) AND ("2010/01/01"[Date - Publication] : "2026/06/30"[Date - Publication])

## Web of Science Core Collection

TS=(("stem cell*" OR "mesenchymal stromal cell*" OR "neural stem cell*" OR "neural progenitor cell*" OR iPSC* OR "embryonic stem cell*" OR "dental pulp stem cell*" OR "extracellular vesicle*" OR "small extracellular vesicle*" OR exosome*) AND ("central nervous system" OR brain OR Alzheimer* OR Parkinson* OR "amyotrophic lateral sclerosis" OR "multiple sclerosis" OR Huntington* OR "frontotemporal dementia" OR "traumatic brain injury" OR "ischemic stroke" OR "spinal cord injury" OR "subarachnoid hemorrhage" OR "optic nerve injury") AND (neuroimmune OR microglia* OR astrocyte* OR inflammasome OR NLRP3 OR "blood-brain barrier" OR senescence OR biodistribution OR pharmacokinetic* OR potency OR biomarker* OR GMP OR nanocarrier* OR biomaterial* OR "clinical trial")) AND PY=(2010-2026)

## Scopus

TITLE-ABS-KEY(("stem cell*" OR "mesenchymal stromal cell*" OR "neural stem cell*" OR "neural progenitor cell*" OR iPSC* OR "embryonic stem cell*" OR "dental pulp stem cell*" OR "extracellular vesicle*" OR "small extracellular vesicle*" OR exosome*) AND ("central nervous system" OR brain OR Alzheimer* OR Parkinson* OR "amyotrophic lateral sclerosis" OR "multiple sclerosis" OR Huntington* OR "frontotemporal dementia" OR "traumatic brain injury" OR "ischemic stroke" OR "spinal cord injury" OR "subarachnoid hemorrhage" OR "optic nerve injury") AND (neuroimmune OR microglia* OR astrocyte* OR inflammasome OR NLRP3 OR "blood-brain barrier" OR senescence OR biodistribution OR pharmacokinetic* OR potency OR biomarker* OR GMP OR nanocarrier* OR biomaterial* OR "clinical trial")) AND PUBYEAR > 2009 AND PUBYEAR < 2027

## ClinicalTrials.gov

(stem cell OR mesenchymal stromal cell OR neural progenitor OR iPSC OR extracellular vesicle OR exosome) AND (Alzheimer disease OR Parkinson disease OR amyotrophic lateral sclerosis OR multiple sclerosis OR Huntington disease OR frontotemporal dementia OR traumatic brain injury OR stroke OR spinal cord injury)

For Web of Science and Scopus, year-based query syntax was combined with screening restricted to records available through June 30, 2026; ClinicalTrials.gov and regulatory records were likewise screened through that date for the literature update.

## Clinical-evidence selection rules

- We prioritized peer-reviewed primary human studies involving direct administration of a stem/progenitor-derived cellular product or EV/sEV preparation in a CNS indication.
- Regulatory records were included when they materially changed translational status (for example, conditional/time-limited authorization).
- Registered but unpublished trials were identified when relevant but were not used as efficacy evidence.
- Safety, target engagement, exploratory secondary outcomes, post hoc subgroup findings, and prespecified efficacy endpoints were kept analytically distinct.
- Non-CNS studies were used only for cross-cutting mechanistic, product-characterization, or delivery principles when directly relevant; they were not used to infer disease-specific clinical efficacy.

# Supplementary Table S1. Study-level application of the neuroimmune niche reprogramming framework

| **Representative study / context** | **Product / route** | **Mechanism specificity** | **Cross-compartment evidence** | **Durability** | **Causal / functional validation** | **Framework interpretation** |
| --- | --- | --- | --- | --- | --- | --- |
| **Wen et al., TBI (31)** | BM-MSC exosomes; systemic | Defined anti-neuroinflammatory EV intervention; pathway-level inflammatory readouts. | Glial/inflammatory and tissue-injury outcomes; limited direct causal communication across compartments. | Short/medium preclinical follow-up; persistence after product clearance not established. | Functional recovery plus inflammatory readouts; limited pathway-ablation evidence. | Level 2 - partial/probable niche remodeling. |
| **Che et al., CNS neuroinflammation (60)** | hUC-MSC exosomes | NRF2/NF-κB/NLRP3-linked mechanism. | Inflammatory and redox/mitochondrial domains engaged; cross-compartment causality incomplete. | Preclinical durability beyond treatment not fully established. | Mechanism-linked molecular and functional effects; not full multi-compartment causal proof. | Level 2 - partial/probable niche remodeling. |
| **Wang et al., SAH (62)** | Olfactory-mucosa MSC exosomes | Mitophagy-linked anti-neuroinflammatory mechanism. | Mitochondrial quality control and inflammatory domains linked; vascular/trophic causality incomplete. | Preclinical follow-up only; post-clearance persistence not established. | Mitophagy-associated rescue with functional readouts. | Level 2 - partial/probable niche remodeling. |
| **Peng et al., PD model (97)** | Engineered therapeutic exosome nanocarrier; intranasal | Route/targeting and cargo mechanism defined. | Target-region exposure plus inflammatory/neuronal outcomes; full cross-compartment causality limited. | Preclinical; long-term state persistence uncertain. | Functional rescue and delivery evidence; pathway perturbation incomplete for whole niche. | Level 2 - partial/probable niche remodeling. |
| **Zhang et al., PD model (98)** | Engineered EV nanoformulation | Defined engineered cargo/immune-homeostasis mechanism. | Immune and neuronal/tissue domains engaged; multicompartment evidence stronger than simple cytokine suppression. | Preclinical durability remains limited. | Mechanism-linked functional rescue; not yet durable causal reprogramming. | Level 2 - partial/probable niche remodeling. |
| **Xie et al., AD human (175)** | Adipose MSC-derived exosome preparation; intranasal | Human product and route defined, but active cargo/causal target not established. | Clinical/cognitive and imaging domains measured; no causal cross-compartment demonstration. | Repeated dosing with follow-up, but persistent tissue-state reprogramming not demonstrated. | Safety/feasibility; exploratory cognitive findings only. | Level 1 - transient/early immunomodulatory or supportive evidence. |
| **Brody et al., AD human (154)** | Allogeneic BM-MSC; IV | Paracrine/immunomodulatory rationale; mechanism not causally isolated. | Clinical, vascular and biomarker domains explored; no causal cross-compartment proof. | Follow-up to 52 wk, but durable niche-state transition not established. | Primary safety met; efficacy exploratory. | Level 1 - immunomodulatory/biomarker evidence. |
| **Schiess et al., PD phase 2 (176)** | Allogeneic BM-MSC; repeated IV | Paracrine/immunomodulatory product; potency heterogeneity is a major unresolved variable. | Motor and inflammatory/biological domains; no direct cross-compartment causal validation. | 88-wk follow-up, but treatment-arm discordance prevents inference of stable reprogramming. | Prespecified clinical comparison with discordant results; mechanism-specific causality not established. | Level 1 - clinical immunomodulatory evidence; no niche-reprogramming proof. |
| **MESEMS, MS (158)** | Autologous BM-MSC; IV | Immunomodulatory rationale. | MRI inflammatory activity and clinical measures, but no coordinated causal state transition. | Crossover follow-up; persistent reprogramming not shown. | Prespecified primary efficacy endpoint neutral. | Level 1 - mechanistic plausibility without clinical reprogramming evidence. |
| **Genchi et al., progressive MS (159)** | Human fetal neural progenitors; intrathecal | Local neuroprotective/immunomodulatory rationale. | CSF/atrophy/clinical domains explored; cross-compartment causal linkage incomplete. | Two-year safety follow-up; durable mechanistic state transition unproven. | Safety/feasibility with exploratory biomarker outcomes. | Level 1-2 - early partial remodeling evidence. |
| **Harris et al., progressive MS (164)** | Autologous MSC-NP; repeated intrathecal | Neuroprotective/immunomodulatory rationale. | Clinical, MRI and CSF domains measured; cross-compartment causal evidence incomplete. | Repeated treatment; durable reprogramming not established. | Primary EDSS Plus endpoint neutral; subgroup findings exploratory. | Level 1 - immunomodulatory/clinical evidence, not coordinated reprogramming. |
| **NurOwn, ALS (160)** | Autologous MSC-NTF; intrathecal | Neurotrophic mechanism/product phenotype defined. | Biomarkers and clinical function assessed; coordinated niche causality not shown. | 28-wk endpoint; durable tissue-state transition unproven. | Primary and secondary efficacy endpoints not met. | Level 1 - target/biomarker activity insufficient for niche-reprogramming claim. |
| **Sawamoto et al., PD (156)** | iPSC-derived dopaminergic progenitors; putaminal graft | Lineage-specific replacement mechanism. | Target engagement/graft survival and motor/PET outcomes; not primarily a niche-reprogramming intervention. | 24-mo graft/safety follow-up. | Direct graft/PET target-engagement evidence; clinical efficacy exploratory. | Replacement-focused; evaluated separately from the niche-reprogramming hierarchy. |
| **Tabar et al., PD (157)** | hESC-derived dopaminergic progenitors; putaminal graft | Lineage-specific replacement mechanism. | PET/graft and motor outcomes; not designed to demonstrate multi-compartment niche reprogramming. | ≥18-mo reported follow-up. | Safety/engraftment evidence; clinical efficacy exploratory. | Replacement-focused; evaluated separately from the niche-reprogramming hierarchy. |
| Rash et al., AD CLEAR-MIND (155) | Laromestrocel/Lomecel-B; IV | Paracrine/immunomodulatory rationale; no causally isolated active mediator. | Clinical, brain-atrophy, and neuroinflammation-imaging domains assessed; no causal cross-compartment linkage. | 39-wk follow-up across single- and repeated-dose regimens; persistent niche-state change after exposure not established. | Randomized placebo-controlled secondary clinical/imaging signals; no pathway perturbation establishing niche causality. | Level 1-2 - early partial remodeling evidence; not causal/durable reprogramming. |
| Schiess et al., PD safety study (35) | Allogeneic BM-MSC; IV | Paracrine/immunomodulatory rationale; dose-escalation product not linked to a validated potency mediator. | Motor and inflammatory readouts explored; no cross-compartment causal demonstration. | 52-wk follow-up; persistence of a mechanistic tissue state after product clearance not established. | Primary safety/tolerability objective met; motor/inflammatory findings exploratory. | Level 1 - clinical immunomodulatory/feasibility evidence; no niche-reprogramming proof. |
| Feldman et al., ALS (161) | Human spinal cord-derived NSCs; intraspinal | Local trophic/neuroprotective and possible lineage-support rationale. | Clinical function and procedure safety assessed; no coordinated multi-compartment niche-state evidence. | Phase I follow-up established procedural safety/feasibility; durable mechanistic state transition not shown. | Dose-escalation safety/feasibility; no efficacy claim and no pathway-ablation evidence. | Level 1 - local supportive/feasibility evidence; not coordinated niche reprogramming. |
| Cox et al., pediatric TBI (162) | Autologous heterogeneous BMMNC fraction; IV within 48 h | Acute injury/immune-repair rationale, but the active cellular mediator is not isolated. | MRI structural and clinical/functional domains were assessed; no direct causal linkage across niche compartments. | Follow-up to 1 y; product persistence and sustained mechanistic state after clearance were not established. | Randomized trial with prespecified MRI endpoints and a white-matter preservation signal; no clear functional-group difference. | Level 1-2 - early partial remodeling evidence; structural signal without causal multi-compartment proof. |
| Cox et al., adult TBI (163) | Autologous heterogeneous BMMNC fraction; IV within 48 h | Inflammatory/structural preservation hypothesis; active cellular mediator not isolated. | MRI and circulating inflammatory readouts were explored; causal cross-compartment linkage incomplete. | Imaging follow-up to approximately 6 mo; durable niche-state transition not demonstrated. | Dose-escalation safety/feasibility established; imaging/cytokine signals exploratory. | Level 1-2 - early partial remodeling evidence; no causal/durable reprogramming. |

Interpretation note: All clinical studies summarized in main-text Table 2 are included below; selected preclinical studies are retained to calibrate the mechanistic levels. Levels 1-4 are descriptive operational categories derived from the manuscript framework, not a validated numerical scoring instrument. Level 1 = transient immunomodulation/supportive evidence; Level 2 = partial/probable niche remodeling; Level 3 = coordinated multi-compartment reprogramming; Level 4 = causal and durable reprogramming. Conservative classification was used; clinical status alone does not increase mechanistic evidence level.
